# A Draft Transcriptome Announcement of *Anguina tritici*

**DOI:** 10.2478/jofnem-2024-0007

**Published:** 2024-03-20

**Authors:** Manish Kumar, Neeraj, Alkesh Hada, Vishal Singh Somvanshi, Ashish Kumar Singh, Yashwant Kumar Yadava, Pradeep Kumar Jain, Kishore Gaikwad, Anil Sirohi

**Affiliations:** Division of Nematology, ICAR-Indian Agricultural Research Institute, Pusa Campus, New Delhi-110012, India; Division of Crop Protection, ICAR-Vivekananda Parvatiya Krishi Anusandhan Sansthan, Almora, Uttarakhand 263601, India; ICAR-National Institute for Plant Biotechnology, Pusa Campus, New Delhi, 110012, India

**Keywords:** *Anguina tritici*, Anhydrobiosis, RNA-seq, Transcriptome, Wheat seed gall nematode

## Abstract

*Anguina tritici*, the wheat seed gall nematode, causes the ‘ear-cockle’ or seed gall disease of wheat (*Triticum sp.*), leading to an extensive decline of yield (30–70%) in underdeveloped wheat cultivating countries of the world. The nematode is known to survive in anhydrobiotic conditions for up to 32 years. Here, we present the first transcriptome assembly of *A. tritici*, which will be a valuable resource for understanding the genes responsible for nematode survival and above-ground plant parasitism. The final 133.2 Mb assembly consists of 105606 open reading frames (including isoforms) with the following BUSCO scores against Nematoda database: 80.3% complete (16.4% single copy and 63.9% duplicated), 2.1% fragmented, and 17.6% missing.

The wheat seed gall nematode, *Anguina tritici*, is a member of the order Tylenchida, which also contains several other plant-parasitic nematodes (Blaxter *et al.,* 1998). Wheat, rye, and barley are the common hosts of *A. tritici* throughout the world, but in India, barley is not frequently attacked (Paruthi and Gupta, 1987). *A. tritici* has six different developmental stages *viz.,* eggs, J1, J2, J3, J4, and adults (both male and female). Second-stage juveniles (J2) are infective and can survive in the anhydrobiotic stage (Gokte and Swarup, 1987). The swollen base of the host plant is the first sign of infection. The awns, glumes, or staminate tissues enlarge or develop tiny bumps, and the glumes become more divergent (Gupta and Swarup, 1968). With the growth of plants, nematode juveniles change their lifestyle (Midha *et al.,* 1971), and once the seed galls mature, J2s undergo anhydrobiosis. The reasons behind the nematode's lifestyle switch to anhydrobiosis and their prolonged survival in anhydrobiotic stage may add new information to the science. This study attempted to generate a transcriptome resource using various developmental stages of *Anguina* nematodes by RNA-sequencing of the anhydrobiotic J2s, revived J2s, and adults.

The white fibrous mass obtained by scraping dry nematode galls served as the source of total RNA extraction from the anhydrobiotic J2s. For the revived J2s, the seed galls were soaked in sterile water for a week, and healthy mobile nematodes were collected for total RNA extraction. The adult stages (male and females both) were collected from the green-colored galls that are formed just before the maturation of the crop. As the seed formation starts, the grains change to green colored galls due to nematode infestation (Gupta and Swarup, 1968). These green galls were collected, cut from a side, and kept in sterile water for 2–3 hours to collect adult nematodes for RNA extraction.

Total RNA was extracted by using a NucleoSpin RNA kit (Macherey-Nagel, Düren, Germany) following the manufacturer's instructions. Total RNA was treated with RNase-Free DNase (Macherey-Nagel, Germany) to remove any gDNA contamination. The quality of RNA was analyzed for its integrity by electrophoresis on a 1.2% (w/v) agarose gel (Sigma, USA) and NanoDrop-2000 spectrophotometer (Thermo Fisher Scientific, USA). Further, the quality of isolated RNA was assessed, and RNA with an RNA Integrity Number (RIN) of more than 8.0 was used for mRNA purification and further subjected to cDNA synthesis. Additionally, the library was prepared by NEBNext® Ultra^TM^ II Directional RNA Library Kit for Illumina. Library integrity was further checked by Qubit 4.0 fluorometer (Thermo Fisher Scientific, USA) and Tapestation 2200 (Agilent Technologies, USA). The cDNA libraries were then sequenced on the Illumina HiSeq platform (NovaSeq 6000) by outsourcing to Bionivid Technologies Pvt. Ltd., Bangalore, India. The raw reads were processed for quality control (QC) by the NGS toolkit, v2.3.3 (Patel and Jain, 2012). The quality filtering and adapter trimming of the raw reads was done using Fastp (ver. 0.20.0) (Chen *et al.,* 2018). A quality Phred score cutoff of 30 was used, and only high-quality reads were retained and used for further downstream analyses. The filtered reads were assembled using Trinity v2.9.1 assembler (Grabherr *et al.,* 2011) at default parameters. Cd-hit-est was used to cluster similar nucleotide sequences within the assembly based on the sequence and length. Transcripts that were 90% identical in sequences and length were grouped together, and only a representative transcript (Unigene) was retained. The three assemblies created from the three developmental stages (anhydrobiotic J2s, revived J2s, and adults) were merged using cd-hit-est (Li & Godzik, 2006) to create a final assembly. The filtered HQ reads were mapped back to final assembly for validation of the assembly by using Kallisto v0.46.1 (Bray *et al.,* 2016). The contaminating sequences were removed by comparing the unigenes against NCBI taxonomy browser. Blobtools (Laetsch and Blaxter, 2017) was used to identify the sequences differing in the GC content and identify potential contaminants. Benchmarking Universal Single-Copy Orthologs (BUSCO) analysis was done for checking assembly completeness (Waterhouse *et al.,* 2018).

Sequencing generated 25–40 (average 29.4) million raw reads per sample. Quality filtering of *Anguina tritici* raw reads yielded about 28.5 million reads for each sample, and the average read length was 151 bp for all the samples. The percentage of HQ reads was more than 97% after quality filtering (Table 1). A total of 139,002 transcripts in anhydrobiotic J2s, 156731 in revived J2s, and 80282 in adult stage of the nematode were identified with N50 length 1403 bp, 1,523 bp, and 1,356 bp, respectively (Table 2). Merging of the reads from the three stages yielded a combined final assembly of 133.2 Mb size comprising 105606 transcripts. The assembly was further validated by mapping the filtered HQ reads from all samples to the final assembly. It was found that 95.04% to 98.63% of the HQ reads mapped back to the final assembly. Blobtools identified that 97.45% reads were mapped, out of which 84.78 % mapped to phylum Nematoda, whereas 12.66% reads had no hits (Figure 1). The BUSCO completeness analysis against Nematoda database showed the presence of 80.3% complete BUSCOs (16.4% single copy and 63.9% duplicated), 2.1% fragmented, and 17.6% missing BUSCOs.

**Table 1.** Raw and filtered read statistics.

**Samples**	**Number of Raw Reads**	**Number of Filtered Reads**	**% HQ Reads**
A1	25538912	24812440	97.2
A2	39242280	38109598	97.1
A3	27283058	26533604	97.3
R1	30749644	29902980	97.2
R2	25571016	24827760	97.1
R3	34695884	33682830	97.1
MF1	29967584	29175744	97.4
MF2	29549536	28787300	97.4
MF3	27678246	26968162	97.4

Samples abbreviations: A – Anhydrobiotic J2; R- Revived J2; MF – Adults.

**Table 2.** Assembly statistics for *Anguina tritici* transcriptome of three developmental stages.

	**Anhydrobiotic J2s**	**Adults**	**Revived J2s**	**Combined Final**
Transcripts	139002	80282	156731	105606
Total bases (Mb)	112.5	65	131.3	133.2
Minimum sequence length (bp)	191	179	187	185
Maximum sequence length (Kb)	24.8	16.7	24.8	24.8
Average sequence length (bp)	809	809	838	1261
N50 length (bp)	1403	1356	1523	1871
(G + C)s	43.27	42.7	41.8	43.2

**Figure 1:**
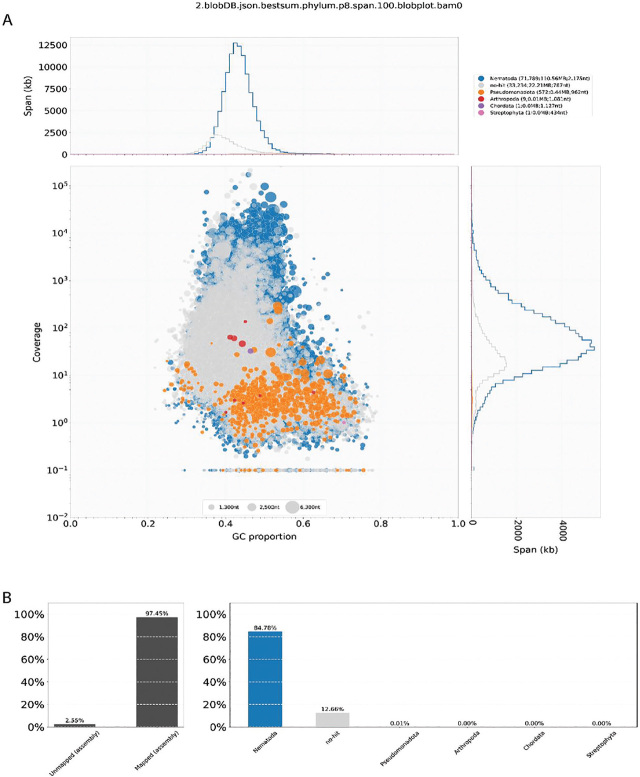
Blob Tool Analysis of Final assembly. (A) Mapped Read GC content (B) Assembly mapping to different phyla.

The draft transcriptome of *A. tritici*. would be useful for the researchers working on comparative genomics of this and other PPNs and would enable functional genomics.

## Data Availability

The transcriptome sequence submitted at NCBI with BioProject ID: PRJNA905225; Bio sample: SAMN31867989, SAMN31867990, SAMN31867991, SAMN31867992, SAMN31867993, SAMN31867994, SAMN31867995, SAMN31867996, SAMN31867997; SRA run accessions SRR22406246, SRR22406245, SRR22406244 (three replicates of anhydrobiotic J2s), SRR22406243, SRR22406242, SRR22406241 (three replicates of revived J2s), SRR22406240, SRR22406239, SRR22406238 (three replicates of adults) and TSA accession GKDZ00000000.
